# A follow-up study of post-COVID-19 syndrome in hospitalized children with Omicron variant infection in Wuhan

**DOI:** 10.3389/fped.2024.1359057

**Published:** 2024-08-01

**Authors:** Chunjiao Tang, Shouyi Wang, Jingyi Fan

**Affiliations:** Department of Pediatrics, Zhongnan Hospital of Wuhan University, Wuhan, China

**Keywords:** COVID-19, post-COVID-19 syndrome, SARS-CoV-2, Omicron variant, hospitalized patients, children

## Abstract

**Background:**

Since the Chinese government changed its COVID-19 prevention and control policies, the rapid spread of the omicron variant resulted in a pervasive surge of infections throughout the nation, particularly affecting children. Although the acute symptoms of children infected with COVID-19 are milder compared to adults, the impact of post-COVID-19 syndromes (PCS) on the growth and development of children should not be ignored. The clinical manifestations, treatment methods, and long-term effects of children are significantly different from those of adults, making it necessary to understand the phenotype of children with PCS in order to effectively manage their health.

**Methods:**

The study focuses on hospitalized children infected with omicron variant in Zhongnan Hospital of Wuhan University from December 7, 2022, to January 5, 2023. Three telephone follow-ups with the guardians was conducted at 4–5 weeks, 12–13 weeks, and 24–25 weeks after the patients' discharge to understand their prevalence, clinical characteristics, and risk factors of PCS.

**Results:**

The age range of the 112 hospitalized pediatric patients was 0–13 years, with a median age of 19 months. After three follow-ups, 49.1% patients had PCS, while the incidence of PCS persisting 3 month was 21.4%, with a prevalence of PCS persisting 6 month of 10.7%. From the first follow-up phase to the third phase, there was a significant decrease in the incidence of PCS. In infants, the most common persistent symptom was sleep disorder (19.2%), followed by respiratory symptoms, diarrhea (8.2%), and decreased appetite (6.8%). In children and adolescents, decreased appetite was the most common persistent symptom (30.8%), followed by respiratory symptoms, fatigue (15.4%), and mood changes (15.4%). Decreased appetite was more common in the children and adolescents, while diarrhea and sleep disorders were more common in the infants. Binary logistic regression analysis and ordered logistic regression analysis showed that times of illness (OR = 1.671, 95% CI: 1.339–2.086) were positively correlated with the duration of symptoms. Times of illness was positively correlated with cough/expectoration (OR = 1.491, 95% CI: 1.039–2.138). Age (OR = 0.844, 95% CI: 0.755–0.944) and re-hospitalization (OR = 0.146, 95% CI: 0.022–0.969) were positively correlated with sleep disorders.

**Conclusions:**

Children with Omicron variant may still experience PCS, but the incidence is lower compared to adults and compared to other variants and the incidence of PCS will gradually decrease over time. The symptoms of PCS differ between older children and infants and it is necessary to prevent recurrent illness for at least half a year after COVID-19 recovery. In order to further understand and ameliorate the impact of PCS on the health of children infected with COVID-19, subsequent follow-up studies will expand the scope, combine with objective follow-up contents, and establish an assessment and management system especially for children of different ages.

## 1 Introduction

Since the end of 2019, an uncommon outbreak of viral pneumonia caused by the SARS-CoV-2, also known as COVID-19, has emerged in Wuhan, China, which is highly contagious (1). As of April 2023, the number of individuals affected by COVID-19 has reached 760 million, resulting in a mortality rate of 6.887 million cases. This is only a conservative estimate, and the actual numbers may be higher.

SARS-CoV-2 has undergone continuous mutations, resulting in the emergence of variants such as Alpha, Beta, Gamma, Delta, and Omicron. These variants have brought about changes in their infectiousness and transmission characteristics (2). Since November 24, 2021, when the South African Ministry of Health reported the Omicron variant to the World Health Organization, the Omicron variant has become the dominant strain of the global COVID-19 pandemic (3). The variant demonstrates heightened transmission velocity and broader dissemination, consequently contributing to a substantial rise in pediatric infection (4).

The initial focus of attention was on the prevalence, hospitalization rate, severity of cases, and mortality rate caused by COVID-19. Over time, people gradually became aware of the long-term effects brought about by COVID-19, which adversely affects both physical and mental health. Although numerous studies have explored the long-term effects of COVID-19 (5, 6), the available data primarily come from adults, while research on the children remains limited.

The post-COVID-19 syndromes (PCS) can either emerge as a new occurrence after recovery from acute COVID-19 or persistently arise from the initial illness. These symptoms may also fluctuate or relapse over time (7). The National Institute for Health and Care Excellence (NICE) in the United States has released guidelines defining PCS as the continuation of symptoms or signs that develop after acute COVID-19. This includes persistent symptoms (lasting 4–12 weeks) and long-COVID (lasting 12 weeks or longer) (8).

On December 7, 2022, the Chinese government changed its COVID-19 prevention and control policies. Discontinuing the enforcement of stringent management and centralized isolation measures in favor of promoting home isolation. Nevertheless, the rapid spread of the omicron variant resulted in a pervasive surge of infections throughout the nation, children as comparative immunity fragile group are more easily infected. Although the acute symptoms of children infected with COVID-19 are milder compared to adults, the impact of PCS on the growth and development of children should not be ignored. The clinical manifestations, treatment methods, and long-term effects of children are significantly different from those of adults, making it necessary to understand the phenotype of children with PCS in order to effectively manage their health.

## 2 Materials and methods

### 2.1 Research subjects

The research subjects for this study consist of pediatric patients infected with the Omicron variant, who were admitted to the pediatric ward of Wuhan University Zhongnan Hospital during the period from December 7, 2022, to January 5, 2023.

Inclusion criteria: (1) Children aged between 0 and 14 years old; (2) Confirmed positive for SARS-CoV-2 nucleic acid; (3) Obtained consent from guardians for a three-phase follow-up.

Age groups: (1) infants group: age <3 years old; (2) children and adolescents group: 3–14 years old.

### 2.2 Follow-up process

#### 2.2.1 Follow-up contents

In our study, as infants are unable to communicate their discomfort and depend on parental observation, only parents of older children were asked about certain aspects during follow-up. We did not ask parents of infants about their general symptoms (fatigue, chest tightness/pain, palpitations, muscles/joints pain, dizziness/headache), neuropsychiatric symptoms (abnormal taste/smell, changes in mood, decreased concentration). Another follow-up contents included vaccination status, parental vaccination status, underlying diseases, secondary infection, times of illness, re-hospitalization. “times of illness” means the times children got sick (with doctor's diagnosis) during the follow-up interval. “duration of exposure” means the time between symptoms onset in the patient and symptoms onset in the first infected person of the same family. The follow-up contents were shown in Tables 1, 2.

**Table 1:** Follow-up contents.

Follow-up contents	Items
Basic information	Underlying medical conditions
	Vaccinations
	Second COVID-19 infection
	Frequency of illness and re-hospitalization
General symptoms	Fever
	Decreased appetite
	Fatigue
	Chest/tightness/pain
	Palpitations
	Muscles/joints pain
	Dizziness/headache
Respiratory symptoms	Cough/expectoration
	Nasal congestion/runny nose
Gastrointestinal symptoms	Abdominal pain
	Diarrhea
	Vomiting
Neuropsychiatric symptoms	Abnormal taste/smell
	Changes in mood
	Decreased concentration
	Sleep disorders (night terrors/restlessness, insomnia, difficulty falling asleep)

**Table 2:** The clinical characteristics of the 112 patients.

Items	Cases (*N* = 112)	Proportion (%)
Gender		
Boy	66	58.9
Girl	46	41.1
Age		
Infants	73	65.2
Children and adolescents	39	34.8
Vaccination status	21	18.8
Parental vaccination status		
ALL	93	83.0
Father/Mother	16	14.3
None	3	2.7
Underlying diseases	18	16.1
Secondary infection	35	31.2
Times of illness		
<5	77	68.8
≥5 and <10	12	10.7
≥10	2	1.7
Re-hospitalization	33	29.5

#### 2.2.2 Follow-up phase

The initial telephone follow-up was conducted at week 4–5 post-diagnosis of SARS-CoV-2. The second telephone follow-up at week 12–13, and a third telephone follow-up at week 24–25. “Phase 1, Phase 2, and Phase 3” means the 3 time points of our follow-up, which defined as the first month, the third month and the 6th month after discharge respectively.

### 2.3 Statistical analysis

Statistical analysis was performed using SPSS 22.0 software. Non-normally distributed continuous data were reported as medians. Categorical data were presented as counts and percentages, and the comparison between groups was analyzed using either the chi-square test or Fisher's exact test. Logistic regression analysis was used to study the independent risk factors of PCS. Differences with *P* < 0.05 were considered statistically significant.

## 3 Results

### 3.1 Clinical characteristics

A total of 121 hospitalized patients with COVID-19 were included in this study. Nine patients were excluded from the study due to incorrect or disconnected phone numbers, and refusal of participation by guardians. In the end, 112 patients were included in the study, resulting in a successful follow-up rate of 92.6%.

Boys (58.9%) outnumber girls (41.1%), with an age range of 0–13 years. The hospitalization rate for infants and toddlers (65.2%) is higher than that for children and adolescents (34.8%), with a median age of 19 months.

The vaccination rate among parents (83%) was higher than that among children (18.8%) (*P* < 0.05). The incidence of reinfection within six months was 31.3% (35, 112), diagnosed based on positive nucleic acid testing or positive antigen testing.

16.1% (18, 112) of the patients had underlying chronic diseases, with the most common being allergic diseases (allergic rhinitis, cough-variant asthma, eczema). Other chronic diseases included congenital adrenal hyperplasia, premature birth, Dravet syndrome, hereditary spherocytosis, chromosomal abnormalities, and autism.

29.5% (33, 112) patients re-hospitalization within six months after discharge. 19.6% (22, 112) patients were readmitted once, 7.1% (8, 112) patients were readmitted twice, and 1.8% (2, 112) patients were readmitted three times. One person hospitalized four times. The main reason for readmission were pneumonia and influenza. The general situation of the 112 patients is shown in Table 2.

### 3.2 Post-COVID-19 syndrome

**Figure 1:**
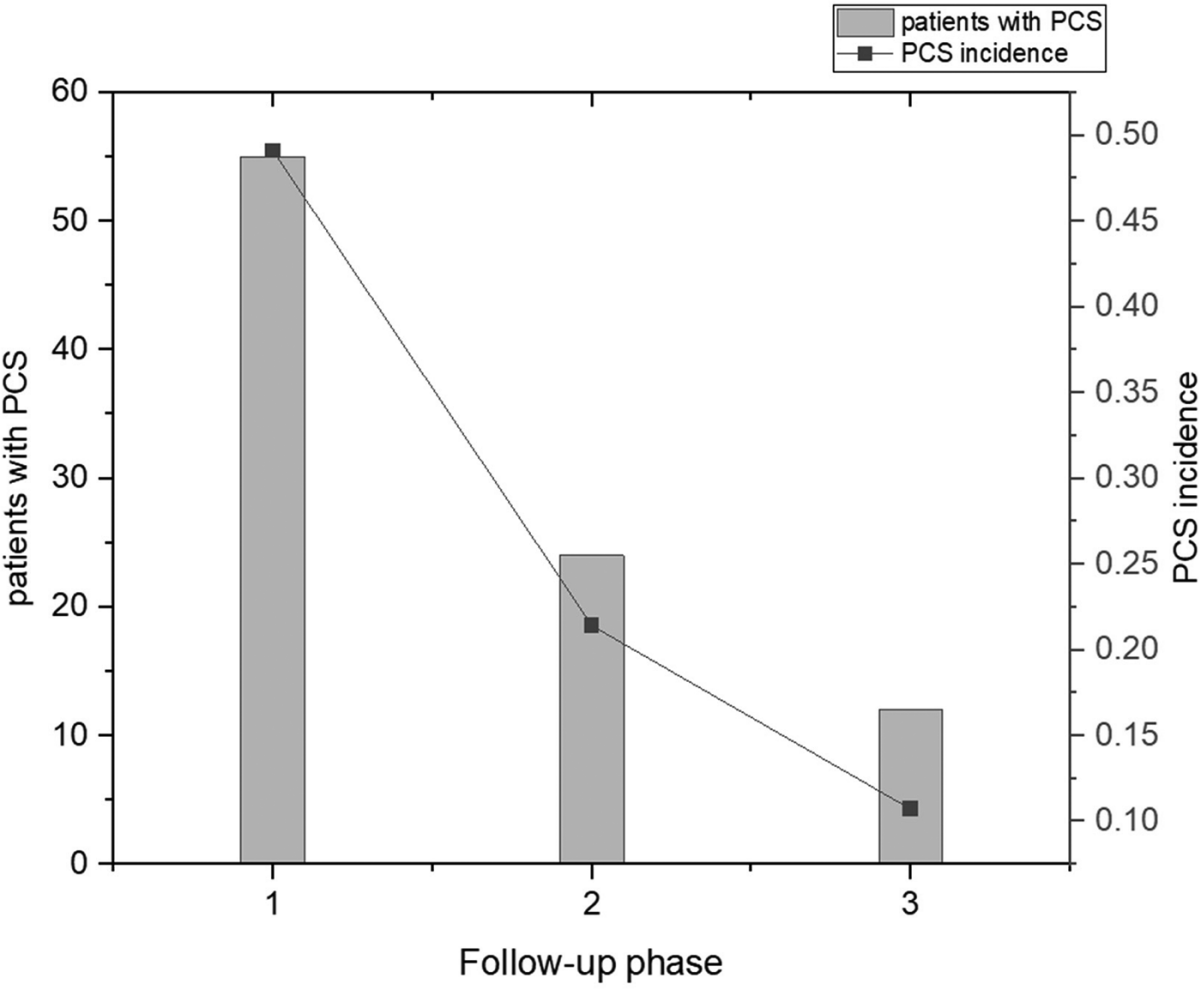
The incidence of PCS at three phases of follow-up.

After three follow-ups, 49.1% (55, 112) of the patients had PCS, with 24.1% (27, 112) experiencing two or more types of PCS. Additionally, 21.4% (24, 112) had at least one symptom that lasted for more than 3 months, while 10.7% (12, 112) had at least one symptom that lasted for more than 6 months. From the first follow-up phase to the third follow-up phase, there was a significant decrease in the incidence of PCS. The incidence of PCS at three phases of follow-up is shown in Figure 1.

Only 1 patient of a 3-month-old baby experienced persistent fever for more than 4 weeks. 15.2% (9/112) patients experienced a decreased appetite, with 8.0% (9, 112) had a decreased appetite for more than 4 weeks, 2.7% (3, 112) for more than 12 weeks, and 4.5% (5, 112) for more than 24 weeks. Decreased appetite was more common in the children and adolescent group.

17.9% (20/112) patients developed persistent cough/expectoration, 20.5% (23/112) patients experienced persistent nasal congestion/runny nose. Persistent cough/expectoration and persistent nasal congestion/runny nose were more common in the infants group.

7.1% (8/112) patients involved abdominal pain/diarrhea, with abdominal pain mainly occurring in children and diarrhea mainly in infants. There were no reported cases of continuous vomiting.

14.3% (16/112) patients experienced sleep disorders, 10.7% (12/112) patients exhibited night fright, 2.7% (3/112) had difficulty falling asleep, and 2.7% (3/112) had nocturnal crying.

Among the 39 cases in children and adolescent group, 15.4% (6/39) experienced fatigue. 6 cases (15.4%) had changes in mood, with 2 cases (5.1%) showing anxiety and 4 cases (10.3%) exhibiting irritability. The characteristics of PCS in different age groups with different follow-up phase was shown in Table 3.

**Table 3:** Characteristics of PCS in different age groups with different follow-up phase.

	Infants (*N* = 73)			Children and adolescents (*N* = 39)		
	Phase 1	Phase 2	Phase 3	Phase 1	Phase 2	Phase 3
General condition						
Fever	1 (1.4)	0	0	0	0	0
Decreased appetite	3 (4.0)	1 (1.4)	1 (1.4)	6 (15.4)	2 (5.1)	4 (10.3)
Fatigue	-	-	-	1 (2.6)	3 (7.7)	2 (5.1)
Chest tightness/pain	-	-	-	2 (5.1)	0	0
Palpitations	-	-	-	0	0	1 (2.6)
Muscles/Joints pain	-	-	-	0	0	0
Dizziness/Headache	-	-	-	0	1 (2.6)	0
Respiratory symptoms						
Cough/Expectoration	12 (16.4)	1 (1.4)	0	3 (7.7)	4 (10.3)	0
Nasal congestion/Runny nose	10 (13.7)	2 (2.7)	1 (1.4)	2 (5.1)	2 (5.1)	6 (15.4)
Gastrointestinal symptoms						
Abdominal pain	0	0	0	2 (5.1)	0	0
Diarrhea	5 (6.8)	1 (1.4)	0	0	0	0
Vomiting	0	0	0	0	0	0
Neuropsychiatric symptoms						
Abnormal taste/smell	-	-	-	1 (2.6)	0	0
Changes in mood	-	-	-	0	0	6 (15.4)
Decreased concentration	-	-	-	0	0	2 (5.1)
Sleep disorders	11 (15.1)	2 (2.7)	1 (1.4)	1 (2.6)	1 (2.6)	0

“-” indicates the contents that are not followed up.

### 3.3 The risk factors analysis of PCS

Gender, age, vaccination status, parental vaccination status, duration of exposure, times of illness, re-hospitalization, secondary infection, and underlying diseases were used as independent variables, and the duration of symptoms lasting for 1 month, 3 months, and 6 months were used as dependent variables for binary logistic regression analysis (see Table 4). The results showed that the duration of exposure (OR = 0.810, 95% CI: 0.684–0.959) was negatively correlated with symptoms lasting for 1 month, while vaccination status (OR = 6.214, 95% CI: 1.171–32.966) and times of illness (OR = 1.854, 95% CI: 1.292–2.661) were positively correlated with symptoms lasting for 1 month. Vaccination status (OR = 10.637, 95% CI: 1.437–78.737) and times of illness (OR = 1.841, 95% CI: 1.313–2.582) were also positively correlated with symptoms lasting for 3 months. The times of illness (OR = 1.424, 95% CI: 1.065–1.903) was positively correlated with symptoms lasting for 6 months. Ordered logistic regression analysis was performed with the duration of symptoms as the dependent variable, and the results indicated that vaccination status (OR = 7.731, 95% CI: 1.873–31.907) and times of illness (OR = 1.671, 95% CI: 1.339–2.086) were positively correlated with the duration of symptoms (see Table 5).

**Table 4:** Binary logistic regression analysis of PCS symptoms duration.

Items	Symptoms persist for 1 month (*n* = 55)		Symptoms persist for 3 months (*n* = 24)		Symptoms persist for 6 months (*n* = 12)	
	*p*-value	OR (95% CI)	*p*-value	OR (95% CI)	*p*-value	OR (95% CI)
Gender	0.420	0.691 (0.281–1.698)	0.157	0.411 (0.120–1.410)	0.476	0.589 (0.137–2.528)
Age	0.124	0.987 (0.970–1.004)	0.603	0.994 (0.971–1.017)	0.695	1.005 (0.980–1.030)
Vaccination status	0.032*	6.214 (1.171–32.966)	0.021*	10.637 (1.437–78.737)	0.577	1.863 (0.209–16.582)
Parental vaccination status	0.578	1.309 (0.507–3.375)	0.200	4.044 (0.477–34.255)	0.762	1.344 (0.198–9.123)
Duration of exposure	0.014*	0.810 (0.684–0.959)	0.729	1.033 (0.861–1.239)	0.670	1.052 (0.833–1.329)
Times of illness	0.001**	1.854 (1.292–2.661)	0.000**	1.841 (1.313–2.582)	0.017*	1.424 (1.065–1.903)
Re-hospitalization	0.081	0.542 (0.272–1.079)	0.607	0.818 (0.381–1.758)	0.977	0.988 (0.416–2.344)
Secondary infection	0.172	1.937 (0.750–5.005)	0.439	1.629 (0.473–5.604)	0.315	2.171 (0.478–9.858)
Underlying diseases	0.757	1.226 (0.337–4.453)	0.091	3.690 (0.810–16.803)	0.057	4.580 (0.958–21.893)

* *p*-value <0.05.

** *p-*value <0.01.

**Table 5:** Ordered logistic regression analysis of PCS symptoms duration.

Items	*p*-value	OR	OR (95% CI)
Gender	0.281	0.653	0.301–1.418
Age	0.126	0.988	0.974–1.003
Duration of exposure	0.005*	7.731	1.873–31.907
Vaccination status	0.458	1.382	0.588–3.248
Parental vaccination status	0.055	0.869	0.753–1.003
Times of illness	0.000*	1.671	1.339–2.086
Re-hospitalization	0.134	0.659	0.381–1.138
Secondary infection	0.110	1.943	0.860–4.392
Underlying diseases	0.108	2.374	0.827–6.808

* *p-*value <0.01.

Symptoms duration, gender, age, vaccination status, parental vaccination status, duration of exposure, times of illness, re-hospitalization, secondary infection, and underlying diseases were used as independent variables, and the PCS symptoms were used as dependent variables for binary logistic regression analysis (see Table 6). The results showed that Symptoms duration was positively correlated with decreased appetite (OR = 3.499, 95% CI: 1.711–7.153), cough/expectoration (OR = 3.345, 95% CI: 1.574–7.107), nasal congestion/runny nose (OR = 4.147, 95% CI: 1.914–8.984) and sleep disorders (OR = 12.633, 95% CI: 2.855–55.889). Times of illness was positively correlated with cough/expectoration (OR = 1.491, 95% CI: 1.039–2.138). Age (OR = 0.844, 95% CI: 0.755–0.944) and re-hospitalization (OR = 0.146, 95% CI: 0.022–0.969) were positively correlated with sleep disorders.

**Table 6:** Binary logistic regression analysis of PCS symptoms.

Items	Decreased appetite		Cough/Expectoration		Nasal congestion/Runny nose		Sleep disorders	
	*p*-value	OR (95% CI)	*p*-value	OR (95% CI)	*p*-value	OR (95% CI)	*p*-value	OR (95% CI)
Symptoms duration	0.001**	3.499 (1.711–7.153)	0.002**	3.345 (1.574–7.107)	0.000**	4.147 (1.914–8.984)	0.001**	12.633 (2.855–55.889)
Age	0.375	1.010 (0.988–1.032)	0.076	0.966 (0.930–1.004)	0.110	0.970 (0.935–1.007)	0.003**	0.844 (0.755–0.944)
Gender	0.500	1.632 (0.393–6.770)	0.305	2.043 (0.522–7.988)	0.603	1.450 (0.357–5.889)	0.564	0.619 (0.121–3.161)
Vaccination status	0.920	0.903 (0.122–6.687)	0.301	3.989 (0.290–54.823)	0.309	3.553 (0.309–40.830)	0.055	224.132 (0.890–56,453.519)
Parental vaccination status	0.157	0.382 (0.101–1.446)	0.645	1.593 (0.220–11.539)	0.896	1.128 (0.185–6.874)	0.276	0.327 (0.044–2.442)
Duration of exposure	0.598	1.076 (0.819–1.414)	0.541	0.935 (0.753–1.160)	0.261	0.844 (0.628–1.134)	0.928	0.988 (0.763–1.280)
Times of illness	0.553	1.100 (0.803–1.507)	0.030*	1.491 (1.039–2.138)	0.057	1.367 (0.991–1.886)	0.907	1.038 (0.552–1.955)
Re-hospitalization	0.971	1.017 (0.419–2.467)	0.657	1.186 (0.558–2.523)	0.783	1.124 (0.488–2.589)	0.046*	0.146 (0.022–0.969)
Secondary infection	0.708	0.748 (0.164–3.417)	0.104	2.914 (0.802–10.591)	0.225	0.386 (0.083–1.797)	0.518	0.579 (0.111–3.030)
Underlying diseases	0.188	2.801 (0.604–12.985)	0.063	0.132 (0.016–1.113)	0.133	3.575 (0.678–18.844)	0.873	0.807 (0.058–11.156)

* *p*-value <0.05.

** *p*-value <0.01.

## 4 Discussion

This study is one of the few studies related to PCS in children, filling the gap in phenotypic research on PCS in children in China. The patients admitted during this period for retrospective study can reflected the adjustment of epidemic prevention and control policy - the infection situation of children after the downgrade of protective measures. In addition, the virus strain of this period is Omicron, which is different from that of 2019. This follow-up study analyzed the prevalence, characteristics, and influencing factors of PCS in 112 hospitalized children infected with the Omicron variant after discharge. Our results showed a prevalence of persistent symptoms in hospitalized children of 49.1%, with a prevalence of long COVID-19 of 21.4%. A study found significant differences in the incidence of PCS in children, ranging from 1.6% to 70%, another study found the incidence of PCS in children was 17.8%-41.9% (9, 10). A large community-based sample size study from Canada found a very low incidence of PCS in children, which was 0.4% (11). The difference in the incidence of PCS between the two studies may be due to different definitions of PCS, different age distributions and different sources of participants. Post–COVID-19 condition (PCC) in Lyndsey et al.’s study was defined as “continuation or development of new symptoms 3 months after the initial SARS-CoV-2 infection, with symptoms lasting for at least 2 months with no other explanation”. Children (aged 8–13 years) in Lyndsey et al.’s study were recruited from the community, whereas our objects were mainly infants (age <3 years) and came from inpatient department. The data on the prevalence of PCS in this study were derived from hospitalized children, and previous studies have shown that hospitalized patients have a higher risk of developing PCS. A meta-analysis showed a prevalence rate of 25.24% for symptoms in non-hospitalized children, while the prevalence rate in hospitalized patients was 29.19% (10). Another global study revealed that the average duration of post-concussion syndrome (PCS) in hospitalized patients was 9 months, contrasting with 4 months in non-hospitalized patients (12). A finding published by Bowe et al. in Nature indicate that two years after infection with the COVID-19, the incidence of PCS in individuals who did not require hospitalization is 31%, while the incidence in hospitalized patients is as high as 65%. In addition, the hospitalized patients still face a significant risk of death (13). This may be because patients who require hospitalization usually have weaker ability to fight the COVID-19, making them more prone to developing severe illness that require treatment, and they also have poorer ability to clear the virus.

A recent epidemiological study on adults in China found that 37.6% of hospitalized patients were diagnosed with PCS (14), while a large-scale data study in Germany indicated that the risk of PCS in children is lower than that in adults, with adults having a 41% higher risk than children (15). One explanation for the higher risk of PCS in adults is that they have more comorbidities, and another explanation is that children have a stronger innate immune system, which gives them stronger antiviral abilities compared to adults (16).

Our results reflect the post-COVID-19 characteristics of children in the context of the epidemic of Omicron variant, while the characteristics of PCS caused by other variants have already been studied. A comparison study in China analyzed the incidence of PCS in hospitalized patients during different variant outbreaks. The results showed that the first-generation variant had the highest incidence of PCS (29.9%–76%), followed by the Delta variant (40.4%), while only 8.89% of patients infected with the Omicron variant reported PCS after discharge (17). A meta-analysis showed that the risk of omicron variant was lower than other variant, and the follow-up time was shorter than others variant (18). According to data from the United Kingdom, the Omicron variant has a 0.24–0.50 reduction in the probability of developing PCS compared with the Delta variant (19). In addition to the characteristics of the Omicron variant itself, such as lower viral replication capacity, this may also be attributed to immunity from vaccination and previous infections.

During the Omicron variant outbreak, we found a higher hospitalization rate in infants (65.2%). A large study in the United States including 14 states also reported the highest cumulative hospitalization rate for children under 2 years old (20). This may be related to the fact that infants and young children have not yet been vaccinated against COVID-19, and vaccination can enhance the clearance of persistent viruses and non-specific immune modulation to reduce hospitalization rates and the occurrence of severe cases (21).

We found that sleep disorders were the most common syndromes in the infant group, followed by respiratory diseases, diarrhea, and decreased appetite. Some studies have also found that sleep disorders are a common syndrome in children (10, 22). But a nationwide large-scale cohort study conducted abroad showed that hospitalized children frequently complained of cough (32.3%) and runny nose (41.9%), with parents of infants reporting more persistent upper respiratory symptoms compared to other age groups (20), Lippi et al. also found that coughing and sneezing were more common in omicron variant with respiratory infections (23). Different results may be related to the sample size. Additionally, symptoms such as coughing and runny nose can be determined by doctor, are the main reasons for hospitalization, but sleep problems are primarily reported by parents and are generally not presented as a primary complaint, which may also lead to result biases.

Our study found decreased appetite was the most common symptom in the children and adolescent group, followed by respiratory symptoms, fatigue, and mood changes. Other studies also have found that older children often report decreased appetite, fatigue (10, 24), and difficulty concentrating (10, 22), which are similar to the symptoms of adults. A study in Germany found that the most common PCS in children were fatigue, cough, and sore throat, while the most common PCS in adults were loss of smell and taste, difficulty breathing, cough, sore throat, chest pain, and hair loss (15). Our research subjects are relatively young, and they generally do not express any olfactory or gustatory abnormalities. However, our research identified a higher incidence of decreased appetite, which could be partly due to olfactory or gustatory abnormalities. In addition, adults are more prone to anxiety, depression, and post-traumatic stress disorder (15, 25, 26), children are also experiencing increasing psychological problems (27), which may be attributed to the isolation and separation from family and friends during quarantine (28), as well as fear of the virus. The cognitive impairment is commonly seen in adults and can also occur in children. It is still unclear whether cognitive impairment will have long-term effects on children's neurological development (29), and further specialized follow-up assessments are needed.

The follow-up results showed that the incidence rate of symptoms decreased with follow-up time. A Danish study noted that at least 54%–75% of patients experienced remission of symptoms within 1–5 months (24). Another report from England also noted that PCS in patients dropped to 1.8% at 8 weeks or more (30). In our study, persistent diarrhea was observed only in infants. Similar to the study conducted by Roge et al., it was found that diarrhea was more common in infants compared to other age groups (22). The gastrointestinal system of infants is immature, with insufficient secretion of digestive enzymes and stomach acid, and the intestinal microbiota is not fully formed. Additionally, the SARA-Cov-2 can disrupt the intestinal microecology and directly invade or cause inflammation damage to the intestinal epithelial cells leading to diarrhea (31).Our binomial logistic analysis found that the younger the easier to develop sleep disorders, because younger children have an immature blood-brain barrier function and a lower immune function, making it easier for viruses and inflammatory mediators to invade the meninges, brain blood vessels, and brain tissue, increasing the risk of meningitis and encephalitis (32).

Based on published findings (33, 34), the respiratory system and nervous system are most affected by long-term COVID-19. For the respiratory system, the airway epithelium is rich in ACE2 (angiotensin converting enzyme2), the target for COVID-19 entry into lung tissue and cause tissue damage (35). Additionally, systemic inflammation caused by COVID-19, such as complement C3 decrease and IL-6, TNF-α increase, are risk factors for long-term lung symptoms and leads to diffuse parenchymal changes in the lungs, including alveolar damage, exudation, and pulmonary fibrosis (36–38). Symptoms may manifest as cough, difficulty breathing, and chest tightness, while functional impairment may present as restrictive or obstructive dysfunction. For the nervous system, it has been demonstrated that the virus can directly invade the olfactory bulb, trigeminal ganglion (39), and cerebral vascular endothelial cells (40), as well as affect the number and activity of glial cells in the brainstem or cerebellum through inflammatory cells or factors (31, 41). This leads to decreased brain metabolism (42), insufficient perfusion, and changes in the structure and functional connectivity of the brain (29), which may explain the long-term central nervous system PCS.

Furthermore, our analysis of influence factors of PCS found a positive correlation between the frequency of illness after infection and the duration of symptoms. During the pandemic, measures such as maintaining social distance, enhancing hand hygiene, and wearing masks were implemented, leading to a lack of immune stimulation in children. Moreover, the resurgence of the virus during the off-season also contributed to the increased susceptibility of children. The immune imbalance caused by the infection with the SARA-Cov-2, such as a decrease in memory T cells (43), excessive activation of cytotoxic T cells and B cells (44), increased pro-inflammatory factors, and the presence of autoantibodies (45), can result in recurrent infections and exacerbate systemic inflammation and tissue damage in affected children.

In ordered logistic regression analysis we did not find the vaccination is associated with the duration of symptoms, we just find that vaccination is correlated with symptoms last for 1 month and 3 months. We did not find any association between underlying diseases, secondary infections, and PCS. Some studies have shown that vaccines can provide partial protection, reducing the risk of PCS by 15%-41% (46). However, some study indicated that receiving one dose of vaccine will not affect PCS (47), and receiving two doses will alleviate PSC. Nevertheless, different variants and previous infections may affect the effectiveness of the vaccine against PCS. One possible reason may be that our sample primarily consists of infants who have not received vaccinations, so we did not observe any correlation between the two; another reason may be that the potency of vaccines against the Omicron variant is weaker compared to other variants (48). Underlying diseases are more associated with an increased risk of severe acute COVID-19 or death, and may also be related to long-term adverse outcomes after the acute phase recovery. One study showed that the shortest interval between initial infection and reinfection is 19 days, while the longest interval is 293 days, secondary or tertiary infections increase the risk of PCS (49). In addition, other studies found that female, older than 40 years, depression, allergic rhinitis, cardiovascular and psychiatric symptoms were positively associated with PCS (50). The findings came mainly from adults and older children, however, our study was mainly infants, psychiatric symptoms and allergic rhinitis could not be assessed by parent-proxy symptom reporting.

## 5 Conclusion

In conclusion, this study is the first single-center study of the PCS in hospitalized children with Omicron variant in Wuhan. Children with Omicron variant who are hospitalized and recover from the acute phase may still experience PCS, but the incidence is lower compared to adults and compared to other variants and the incidence of PCS will gradually decrease over time. With different spectrum of PCS, infants are more prone to diarrhea and sleep disorders, and the younger the more likely to develop sleep disorders, children and adolescents are more prone to decreased appetite. Limitations of our study include a small sample size, depending on parent-proxy symptom reporting and the narrow age range of participants.

Further research will focus on broadening the follow-up cohorts to include children of all ages and is necessary to investigate the neurobehavioral consequences of SARS-CoV-2 infection. In the future, we need to establish an evaluation management system that includes prevention, treatment and follow-up to facilitate in-depth research on the long-term impact of COVID-19 on children's health.

## Data Availability

The original contributions presented in the study are included in the article/Supplementary Material, further inquiries can be directed to the corresponding authors.
